# Surgical Treatment of Intra-Abdominal Desmoid Tumors Resulting In Short Bowel Syndrome

**DOI:** 10.3390/cancers4010031

**Published:** 2012-01-19

**Authors:** Matthew Wheeler, David Mercer, Wendy Grant, Jean Botha, Alan Langnas, Jon Thompson

**Affiliations:** Department of Surgery, University of Nebraska Medical Center, The Nebraska Medical Center 3280, Omaha, NE 68198, USA; E-Mails: mjwheele@unmc.edu (M.W.); dmercer@unmc.edu (D.M.); wgrant@unmc.edu (W.G.); jbotha@unmc.edu (J.B.); alangnas@unmc.edu (A.L.)

**Keywords:** intra-abdominal desmoid tumors, short bowel syndrome, familial adenomatous polyposis

## Abstract

Advanced intra-abdominal desmoids tumors present with severe symptoms, complications or rapid growth, which lead to adverse outcomes. Our aim was to evaluate the treatment and outcome of patients with advanced intra-abdominal desmoids tumors, and develop guidelines for surgical management of these patients. We reviewed the clinical courses of 21 adult patients with advanced stage intra-abdominal desmoid tumors who presented to an intestinal rehabilitation and transplantation program. Patients with massive intestinal resection presented in two groups. The first group had a short small intestinal remnant after resection (<60 cm). These patients were poor rehabilitation candidates and eventually met criteria for transplant. The second had longer intestinal remnants and were more successfully rehabilitated and have not had complications that would lead to transplantation. Advanced intra-abdominal desmoid tumors have outcomes after resection that merit aggressive resection and planned intestinal rehabilitation and intestinal transplantation as indicated.

## 1. Introduction

Intra-abdominal desmoid tumors, also known as aggressive fibromatosis, are a rare and complex problem. The incidence is 7/100,000 among the general population. Sporadic cases make up 80% of intra-abdominal desmoids tumors while 20% are associated with patients with Familial Adenomatous Polyposis (FAP) [1]. Among FAP patients, 10%–20% present with desmoids tumors sometime during their disease course. The FAP patients with intra-abdominal desmoids present at a young age, often before the fourth decade of life and are more likely to have advanced or complicated disease [2,3]. The sporadic tumors have been demonstrated to be less aggressive. This difference is reflected by the higher mortality and recurrence rate of intra-abdominal desmoids tumors in FAP patients in comparison to patients without FAP [4,5]. Sporadic cases of intra-abdominal desmoids are less commonly advanced tumors presenting with complications or requiring resection.

These tumors are usually treated by a multimodality approach. Determining medical therapy is difficult: the tumors follow a variable course and few well-structured trials exist in these rare tumors. In many patients tumor growth can be suppressed with non-steroidal anti-inflammatory drugs (NSAIDs) or therapy with anti-estrogen drugs (tamoxifen, raloxifene or toremifene). Chemotherapy agents such as doxorubicin, adriamycin, methotrexate, velban, sorafenib and Gleevec have been used in more advanced or progressive tumors with limited success [6,7]. There are currently ongoing trials for external beam radiation and intraoperative radiation for patients in whom resection with negative margins would require massive resection [8,9]. However, radiation therapy has not been used extensively in intra-abdominal desmoid tumor patients. These modalities offer promise for patients with slow growing sporadic desmoids. However, patients with complicated intra-abdominal desmoids and FAP have aggressive disease that requires aggressive treatment. These patients often present with significant amounts of bowel to be resected or with severe complications, such as obstruction, peritonitis or ischemia which require intervention for their survival, rather than simply for relieving symptoms.

The Collaborative Group of the Americas on Inherited Colon Cancer has developed a staging and treatment system that stratifies intra-abdominal desmoids tumors based on prognosis. This staging system integrates size (in largest diameter), symptoms, and growth rate to predict what the prognosis is and what treatment should be applied [10]. As seen in Table 1, small (<10 cm), stable, and asymptomatic tumors can be watched (Stage I); small, symptomatic tumors should be resected (Stage II) when feasible; large (10–20 cm) and symptomatic, or slow growing (<50% increase in 6 mo) and asymptomatic tumors require medical therapy (Stage III); and large (>20 cm) or rapidly growing or complicated desmoid tumors are likely to require radical resection and urgent intervention (Stage IV). A retrospective review of FAP associated intra-abdominal desmoids at the Cleveland Clinic validated this system, noting progressively worsened outcomes with advancing stage [11].

The difficulty in decision making for timing and type of surgery in higher stage disease is the the need to radically resect small intestine and the presence of symptoms and complications. A consequence of massive resection of mesenteric desmoids tumors is short bowel syndrome (SBS). SBS in adults is defined as less than 200 cm of small intestine remaining with symptoms of malabsorption [12]. These patients often require care at intestinal rehabilitation centers that function in multidisciplinary teams using medical and surgical therapy to maximize nutrition and enteral function. This approach should minimize symptoms and complications, assist in intestinal adaptation, and promote independence from parenteral nutrition (PN). Patients with unresected desmoids fare as well on home PN as other groups of patients with intestinal failure from other etiologies, and have similar complications [13]. For many patients, particularly those with intestinal remnants less than 60 cm, nutritional management in SBS patients is long term PN. Experience in intestinal rehabilitation has demonstrated that adult patients with less than 60 cm of intestine remaining are more likely to remain PN dependent, more likely to develop PN related liver disease, and thus have a higher mortality rate. Intestinal transplantation is an important modality to consider when patients fail intestinal rehabilitation, but the timing and selection are controversial. Since FAP patients make up most of this advanced intra-abdominal desmoids, these patients often present for intestinal rehabilitation late in the course of their disease: they have undergone colectomy, have a history of sepsis and other complications from PN. Our hypothesis was the length of intestinal remnant would be an important determinant of management and outcome. Our aim was to evaluate the treatment and outcome of patients with advanced intra-abdominal desmoids tumors at an intestinal rehabilitation program, and develop guidelines for surgical management of these patients.

**Table 1:** Clinical staging of intra-abdominal desmoids.

Stage	Size	Symptoms	Growth	Treatment Recommendation
I	<10 cm	Asymptomatic	Stable	Observation ± NSAIDs
II	<10 cm	Mild Symptoms	Stable	NSAIDs ± anti-estrogen drugs, Resection
III	10–20 cm	Moderate Symptoms	Slow Growing	NSAIDs + anti-estrogen drugs, Cytotoxic Therapy
IV	>20 cm	Severe Symptoms / Complications	Rapid Growing	Resection

Table 1 was adapted from Church *et al.* [10].

## 2. Results and Discussion

Three groups of patients with similar clinical courses emerged after review. The first group of patients comprised those who did not receive a resection of their intra-abdominal desmoids tumors. The second, included those who had a short remnant of small bowel after resection (<60 cm) and had poor outcomes, requiring evaluation for intestinal transplant. The third group of patients were those patients that had SBS with an adequate remnant (60–200 cm) and underwent intestinal rehabilitation. Only two of 21 patients with advanced stage intra-abdominal desmoids did not have FAP, both of whom survived and gained enteral independence.

Three patients underwent consultation for mesenteric desmoid tumors without receiving a resection. The first of these patients presented with obstruction after colectomy and ileal pouch procedure. It was determined that she would be left with a short segment of small intestine, incompatible with rehabilitation, before the era of intestinal transplantation. She underwent intra-operative radiation therapy with implantation of radiation seeds, in lieu of aggressive resection. She was doing well at last follow up four years after surgery. The second patient was an unfortunate woman who experienced rapid growth of desmoids tumors during pregnancy. She developed small bowel obstruction and sepsis, and died before resection, within weeks of presentation. A third patient had asymptomatic desmoids noted at the time of proctocolectomy. These masses progressed, eventually causing fistula, abscess, and intestinal failure. The patient had percutaneous drainage and is being evaluated for resection.

Nine patients underwent resection leaving a small bowel remnant that predicted successful intestinal rehabilitation. The median (range) remnant length was 125 (73–180) cm and median (range) follow up was 27 (1–161) months. All patients are alive. None of these patients developed a complication of SBS that creates an indication for intestinal transplant. Six patients are independent of PN. Three patients who remain PN dependent are at 1, 22 and 94 months after initial consultation. These patients had a small bowel remnant of 73, 95 and 135 cm, and are continuing intestinal rehabilitation. None of these patients has had a known recurrence since curative resection, though one had abdominal sepsis in the perioperative period requiring reoperation.

Nine patients underwent resections that left an inadequate small intestine length for intestinal rehabilitation, all with segments of small intestine less than 60 cm. The median (range) follow up of these patients is 9 (3–59) mo. All nine patients have been evaluated for transplantation. Four patients died of complications of liver disease (3) and line sepsis (1) prior to intestinal transplantation. One patient is being evaluated after resection and is PN dependent. Four patients received transplants that included intestinal grafts: Two patients died suddenly less than 90 days after transplant one small bowel transplant and a Liver, Small Bowel, Pancreas transplant. Two patients survived the perioperative period: one isolated small bowel transplant and one liver, small bowel, pancreas transplant. Both of these patients are doing well and are on enteral nutrition 9 mo and 4 years after transplantation, respectively. Of interest, one of these patients had been initially prepared for evisceration, *ex vivo* resection, and autotransplantation; but her disease was not amenable and she underwent a near total enterectomy. Only 3 of these 9 patients are still alive.

Patients with advanced intra-abdominal desmoids tumors are a challenging group of patients. These tumors can cause significant complications which will be fatal if not resected. However; extensive resection has high mortality and morbidity and leads to SBS. Mortality as a direct result of intra-abdominal desmoids in series of FAP patients ranges from 18%–53%. In patients without FAP desmoid related mortality is lower, 10%–27% [4,5,14,15]. In our series of patients with advanced stage intra-abdominal desmoid tumors the overall mortality was 28%, which is comparable to patients in other series [11]. No patients who received surgical resection of their tumors died of desmoid related disease, but these deaths occurred as a result of SBS: PN induced liver failure, or sepsis from line access. Of the surviving patients 86% are off PN. We have not found any recurrence in patients operated on with complete resection of their tumors. Most of the patients in this series have FAP and have previously undergone colectomy, making these patients with SBS more difficult to manage. The patients with sporadic intra-abdominal desmoids appear to have good outcomes: both had remnants greater than 60 cm and good recovery from resection.

While conventional surgical approaches would suggest that many advanced intra-abdominal desmoids tumors are unresectable, we found only a small proportion that were clear candidates for palliative management. This is usually determined by the patient’s overall medical condition and complications related to the desmoids to the tumor. We managed to salvage several patients who were previously considered unresectable.

Intra-abdominal desmoids tumors can lead to an emergent operation for complete bowel obstruction, intestinal ischemia, or perforation with peritonitis. In these situations exploration must be undertaken to deal with the acute surgical problem. The patients who survive these treatments can then be considered for definitive treatment of their tumors.

We found that one half of our patients who had undergone resection of their tumor with associated massive bowel resection had a sufficient intestinal remnant to predict successful intestinal rehabilitation with transition to enteral feeding only. This generally requires a small intestine remnant greater than 60 cm since most patients have had previous colectomy due to FAP. Two thirds of this group are now independent of PN and free of tumor. Evisceration, *ex vivo* dissection, and intestinal auto-transplantation is a novel approach that may help preserve enteral independence in selected patients [16]. Our only attempt at this approach was not feasible.

The other group of our patients had undergone resection of their tumor and were left with intestinal remnants less than 60 cm. This length predicts PN dependence with a significantly increased risk of mortality secondary to sepsis and liver disease. Thus, these patients warrant evaluation for intestinal transplantation. Multivisceral transplantation may be appropriate if multiple organs are involved by the tumor or if complications of liver disease have developed. The poor outcome of these patients on PN has led some centers to consider simultaneous intestinal or multivisceral transplantation at the time of resection. However, many patients at our center had already undergone resection prior to referral.

The role of intestinal and multivisceral transplant for patients with complicated abdominal desmoid tumors who fail intestinal rehabilitation is evolving. Our center had the means to provide patients with an opportunity for survival after their disease left them with a short intestinal remnant. Cruz and Abu-Elmagd *et al.* reported a case series of 10 patients who received modified multivisceral transplants (stomach, pancreas and intestine) with 80% long term survival and no recurrences [17]. The groups at Miami and Mount Sinai report more than 80% enteral independence and one recurrence in patients who have disease that precludes autotransplantation [18]. However, other transplant groups have identified very poor outcomes for transplantation with complicated intra-abdominal desmoids, reporting very high mortality and poor graft survival in patients who received allografts that include intestine [19]. The widely accepted indications for intestinal transplant (failure of parenteral nutrition) are currently liver failure secondary to TPN, loss of central venous access, sepsis related to lines, recurrent line infections [20]. The high mortality in patients evaluated and awaiting intestinal transplantation in our experience underscores the importance of early referral. Also, the deaths observed in patients on the waiting list may support the consideration of simultaneous transplant with resection when feasible.

**Table 2:** Summary of outcomes in patients with advanced intra-abdominal desmoid tumors.

Group	Patients	Median Intestinal Length (Range)	FAP	Mortality	Enteral Independence
Unresected	3	-	3/3	1/3	1/2 (surviving)
Intestine <60 cm remaining	9	125 (73–180)	9/9	0/9	6/9
Intestine >60 cm remaining	9	<30	7/9	6/9	2/3 (surviving)
Total	21	-	19/21	7/21	9/14 (surviving)

## 3. Experimental Section

A retrospective review of 21 adult patients (>19 years of age) with SBS secondary to intra-abdominal desmoids was conducted. These patients represent all evaluations by surgeons in the practice of intestinal rehabilitation or intestinal transplantation at University of Nebraska Medical Center (UNMC) over a 23 year period. SBS was defined as less than 200 cm of small intestine with symptoms of malabsorption. Patient data was recorded with emphasis placed on surgical and medical treatment of desmoid tumors, history of FAP and related manifestations, remnant bowel length, and nutritional outcomes. Follow up time is recorded from initial consultation. All research was conducted on a protocol approved by the Institutional Review Board at UNMC.

The patient group included 11 females and 10 males. Median (range) age at surgical consultation was 33 (17–70) years. Median (range) follow up was 27 (1–97) months. 90% (19) patients had a diagnosis of FAP, and of those 89% (17) had undergone colectomy. All of the patients presented with Cleveland Clinic Grade IV disease having large tumors complicated by either severe symptomatic obstruction or fistula with history of abscess. Twelve patients presented with clearly documented failure of medical therapy, with progression of tumor and the occurrence of complications requiring surgery while on these treatments. All patients with documented medical therapy received NSAIDs. Eight patients received NSAIDs in combination with anti-estrogen drugs. After failure of these regimens, six patients received chemotherapy with taxol, adriamycin or Gleevec and one received radiation in an attempt to slow tumor progression.

## 4. Conclusions

**Figure 1:**
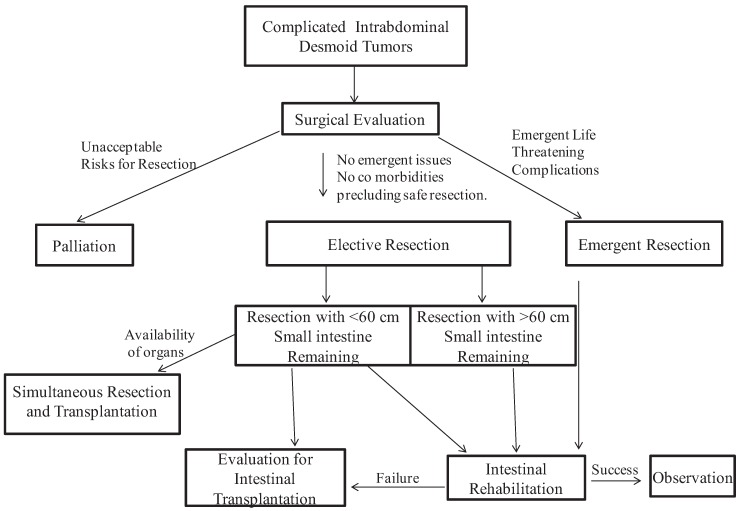
Treatment algorithm for the surgical management of complicated intra-abdominal desmoids tumors.

We propose a treatment algorithm (Figure 1) for surgical management of advanced intra-abdominal desmoid tumors utilizing aggressive surgical approaches.

This algorithm begins with development of complications requiring surgery. There will remain a few individuals who are truly unresectable and should have palliative treatment. These patients include those that are unlikely to survive operations or unable. If the patient has disease threatening significant portions of small bowel evaluation at centers offering intestinal rehabilitation and transplantation should be part of the decision making. Any patient that can be adequately resected without leaving a very short remnant should undergo surgery with intestinal rehabilitation. The patients that are left with a very short remnant are a difficult group and need to be referred for intestinal transplant. The concept of simultaneous transplant at the time of resection requires further evaluation. Multivisceral transplantation will be appropriate in selected patients. We believe this surgical management scheme will give optimal results in this challenging group of patients with advanced intra-abdominal desmoids tumors.

## References

[1] Burke A.P., Sobin L.H., Shekitka K.M., Federspiel B.H., Helwig E.B. (1990). Intra-abdominal fibromatosis. A pathologic analysis of 130 tumors with comparison of clinical subgroups. Am. J. Surg. Pathol..

[2] Nieuwenhuis M.H., Lefevre J.H., Bulow S., Jarvinen H., Bertario L., Kerneis S., Parc Y., Vasen H.F. (2011). Family history, surgery, and APC mutation are risk factors for desmoid tumors in familial adenomatous polyposis: An international cohort study. Dis. Colon Rectum.

[3] Melis M., Zager J.S., Sondak V.K. (2008). Multimodality management of desmoid tumors: How important is a negative surgical margin?. J. Surg. Oncol..

[4] Lev D., Kotilingam D., Wei C., Ballo M.T., Zagars G.K., Pisters P.W., Lazar A.A., Patel S.R., Benjamin R.S., Pollock R.E. (2007). Optimizing treatment of desmoid tumors. J. Clin. Oncol..

[5] Smith A.J., Lewis J.J., Merchant N.B., Leung D.H., Woodruff J.M., Brennan M.F. (2000). Surgical management of intra-abdominal desmoid tumours. Br. J. Surg..

[6] Okuno S. (2006). The enigma of desmoid tumors. Curr. Treat. Options Oncol..

[7] Okuno S.H., Edmonson J.H. (2003). Combination chemotherapy for desmoid tumors. Cancer.

[8] Roeder F., Timke C., Oertel S., Hensley F.W., Bischof M., Muenter M.W., Weitz J., Buchler M.W., Lehner B., Debus J. (2010). Intraoperative electron radiotherapy for the management of aggressive fibromatosis. Int. J. Radiat. Oncol. Biol. Phys..

[9] Pajares B., Torres E., Jimenez B., Sevilla I., Rodriguez A., Rico J.M., Trigo J.M., Alba E. (2011). Multimodal treatment of desmoid tumours: The significance of local control. Clin. Transl. Oncol..

[10] Church J., Berk T., Boman B.M., Guillem J., Lynch C., Lynch P., Rodriguez-Bigas M., Rusin L., Weber T., Collaborative Group of the Americas on Inherited Colorectal Cancer (2005). Staging intra-abdominal desmoid tumors in familial adenomatous polyposis: A search for a uniform approach to a troubling disease. Dis. Colon Rectum.

[11] Church J., Lynch C., Neary P., LaGuardia L., Elayi E. (2008). A Desmoid tumor-staging system separates patients with intra-abdominal, familial adenomatous polyposis-associated desmoid disease by behavior and prognosis. Dis. Colon Rectum.

[12] Thompson J.S., Weseman R., Rochling F.A., Mercer D.F. (2011). Current management of the short bowel syndrome. Surg. Clin. North Am..

[13] Shatnawei A., Hamilton C., Quintini C., Steiger E., Kirby D.F. (2010). Use of home parenteral nutrition in patients with intra-abdominal desmoid tumors. Nutr. Clin. Pract..

[14] Berk T., Cohen Z., McLeod R.S., Stern H.S. (1992). Management of mesenteric desmoid tumours in familial adenomatous polyposis. Can. J. Surg..

[15] Clark S.K., Neale K.F., Landgrebe J.C., Phillips R.K. (1999). Desmoid tumours complicating familial adenomatous polyposis. Br. J. Surg..

[16] Tzakis A.G., de Faria W., Angelis M., Verzaro R., Pinna A. (2000). Partial abdominal exenteration, *ex vivo* resection of a large mesenteric fibroma, and successful orthotopic intestinal autotransplantation. Surgery.

[17] Cruz R.J., Costa G., Bond G.J., Soltys K., Rubin E., Humar A., Abu-Elmagd K.M. (2011). Modified multivisceral transplantation with spleen-preserving pancreaticoduodenectomy for patients with familial adenomatous polyposis “Gardner’s Syndrome”. Transplantation.

[18] Chatzipetrou M.A., Tzakis A.G., Pinna A.D., Kato T., Misiakos E.P., Tsaroucha A.K., Weppler D., Ruiz P., Berho M., Fishbein T. (2001). Intestinal transplantation for the treatment of desmoid tumors associated with familial adenomatous polyposis. Surgery.

[19] Lauro A., Zanfi C., Pelligrini S., Cantena F., Pironi L., Pinna A. (2011). Timing of Intestinal/Multivisceral Transplantantation for Gardner’s Syndrome on Adults: Review of a Single Center Experience. Presentation at ISBTS 2011 Symposium.

[20] Fishbein T.M. (2009). Intestinal transplantation. N. Engl. J. Med..

